# Monitoring trends in psychosocial and physical working conditions: Challenges and suggestions for the 21^st^ century

**DOI:** 10.5271/sjweh.3973

**Published:** 2021-06-29

**Authors:** Hermann Burr

**Affiliations:** Bundesanstalt für Arbeitsschutz und Arbeitsmedizin (BAuA) Nöldnerstraße 40-42, 10317 Berlin, Germany Email: burr.hermann@baua.bund.de

In work and health research, there is a lack of studies on prevalence of psychosocial (eg, quantitative demands, social relations) and physical (eg, physical activity, heavy lifting) working conditions among national employee populations – and their trends. [In the following, I shall not discuss the issue of trends of other exposures, such as chemical and dust exposures (1).] To my knowledge, in the recent decade, only a few studies have investigated this topic (2–8), and, in this issue of the *Scandinavian Journal of Work, Environment and Health*, such a rare study is published (9).

## Trends

This issue of the journal includes a new Swedish study, which not only aims to distinguish between different types of macro trends in working conditions (going beyond the assumption of simple linear trends) but also examines whether the gap between good and bad – in terms of working environment – jobs has widened. The reason why it makes sense to go beyond trends in working conditions is obvious. Regarding the industrialized countries, some findings indicate that working conditions are largely deteriorating (3, 5, 8). Other findings indicate that inequalities in distributions of working conditions are increasing (10, 11). A third group of findings suggest that no uniform trends exist; trends, if any, are different from country to country (7) . It has been suggested that the reason for these trends (deterioriation of or increased inequality in exposure to working conditions) might be found in the last four decades of liberalization of labor markets accompanied by globalization and digitalization (10–12). Corin et al’s study (9) in this issue of the journal, however, neither shows a downward uniform trend nor an increased inequality in quality of working conditions in Sweden. However, the question remains, if monitoring data in other countries were analyzed in the same manner as in this new Swedish study, what trends would we see in these countries?

## Reasons for lack of scientific research focus

One can wonder why there are comparably few studies on trends in working conditions. In his first edition of *Modern Epidemiology*, Rothman claims that researchers of health risk factors tend to jump directly to advanced regression analyses skipping a thorough inspection of their data, including which groups in the analysed population are exposed to what exposures (13). Simple intercorrelation tables, or even the traditional ‘Table 1’ showing independent variables broken down by gender or socioeconomic status might be insufficient. Regarding a range of work environment factors, prevalences by occupation can give rich information on what a given exposure actually means for the worker. Let me just give an example from Denmark, a labor market with working hour restrictions especially among blue-collar workers negotiated by the social partners (14, 15). If we take the dimension ‘work–family conflict’, many would immediately think of the overworked white-collar worker with deadlines, eg, journalists, managers, or academics. But the occupational groups reporting the highest level of this conflict in Denmark are not these groups but rather truck drivers (an occupation directly subject to competition from peers from less regulated labor markets), health workers (including only one sole group of academics, namely medical doctors) and other workers subjected to night work (16). In order to understand what a variable really measures, inspection of occupational patterns can be invaluable for both practitioners and researchers. Even a lack of variation due to occupation can widen the understanding of what a variable measures. This applies, for example, to quality of leadership or social support (17, 18). These variables are assumed to rather reflect traits of organizations and departments than traits of occupations (19). So, even if research data in most cases serve purposes other than monitoring, it is worthwhile collecting and classifying data on occupation (even if it is costly) – or at least consulting local monitoring data – in order to understand better what working conditions measure.

## Working conditions: Monitoring trends versus investigating risk factors

It is striking that monitoring data in general are collected in organizational settings other than those where data are used for research on associations between work, health and labor market participation (20, 21). Also, requirements of monitoring data are quite different from requirements of research data. In order to measure long-term trends, monitoring data should collect the same data over time. Only if new issues arise, old issues disappear, or profound methodological issues emerge, should changes in monitoring be introduced. One such methodological issue is the decreasing trend in participation in surveys (22). Researchers who, on the other hand, are looking at work as a possible predictor of health or labor market participation, tend to improve measurements and introduce new risk factors [therefore, the IPD-Work consortium approach of pooling data to overcome limited power of a majority of research datasets is challenged by deviating measurements of risk factors (23, 24)]. However, this does not mean that monitoring data are more conservative and research data more innovative. A comparison of psychosocial content of European monitoring questionnaires reveals a much wider focus on psychosocial factors than the one employed in longitudinal research of cardiovascular disease, burnout or depressive symptoms (25, 26). Note that the distinction of the labels ‘monitoring’ and ‘research’ assumes that dealing with prevalence and trends in working conditions is not research, an assumption which Corin et al’s paper in this issue of the journal refutes.

## Two challenges: Precariousness and border crossing

In industrialized countries, most data on work and health are collected among workers employed as wage earners working in workplaces in their country of permanent residence. This restriction is challenged by (i) various types of precarious employment contracts and (ii) work arrangements crossing borders, ie, involving a dislocation of work from place of residence.

Precarious employment contracts have been on the rise in recent decades and comprise, for example, temporary contracts (some very short term), no contract (including illegal schemes), self-employed one person employment and crowd work (27–30). Note that the prevalence of types of precarious work contracts differs very much from country to country (31). Workers on such contracts might be harder to reach in surveys. In some cases, they might be excluded from sampling frame definitions if the data provider (eg, the state or the company) does not register them as employees. Of course this problem does not arise in studies where the sample frame is based on resident populations allowing also for inclusion of workers not classified as such in registers (2–9). If however precarious workers are contacted in surveys, their uncertain or temporary (or even illegal) situation could make it difficult for them to consider themselves as employees and describe their work situation.

Work arrangements crossing borders have also increased over the last decades (32, 33). This can involve – in its more simple form – border crossers, ie, workers living in one country working in another crossing borders on a daily basis (34). Such arrangements comprise among other things also posted workers [ie, those being sent by their company to work in another country on a temporary basis; this applies to workers in construction companies who are brought from the companies’ country of location to the country where construction takes place (32, 35, 36)]. Another arrangement includes temporary migrant workers who work for a short period of time in agriculture, eg, harvest seasons or those who are employed as nannies, nurses or truck drivers in periods of several years (37, 38). Such temporary workers might seek work in another country on their own, they might be hired by the company they work for in their home country, or a third party might organize the work arrangement, which again can be formal, informal or illegal (39). Also workers crossing borders might be difficult to reach in surveys. They might not be part of sampling frames in the country – or company – where work is carried out (39). Moreover, when interviews are carried out, language barriers could hamper survey participation. To complicate things further aspects of precariousness and border crossing can flow together (eg, illegal work arrangements and residence), further complicating data collection.

In some countries and sectors, these challenges (precariousness and border crossing) might play a role for the coverage of existing monitoring systems or research in work and health. Appropriate data collection and analytical methods exist and should be applied to meet these two challenges (40, 41).

## Concluding remarks

After this, two important questions remain: (i) Do macro trends in working conditions show continuous improvements or deteriorations over time? (ii) Is there is a widening of inequalities in unfavourable working conditions, both within and between occupations?

In many industrialized countries, monitoring data are available that can help answer these questions (20). For some working conditions, monitoring data over time are readily available in population and workforce- based surveys, whereas, for other work-related risk factors, data on trends over time are lacking. At least regarding European surveys, it seems that psychosocial factors are covered more broadly than physical factors (26, 42). Knowledge on trends are of paramount importance for (i) identification of new risk factors, (ii) determining whether occupational health interventions on the national level are successful, and (iii) the quantification of the health impact of various hazards. I hope that the present issue’s paper by Corin et al will lead to more contributions within this field of research.
